# Web-Based Cognitive Testing of Older Adults in Person Versus at Home: Within-Subjects Comparison Study

**DOI:** 10.2196/23384

**Published:** 2021-02-01

**Authors:** Andrée-Ann Cyr, Kristoffer Romero, Laura Galin-Corini

**Affiliations:** 1 Department of Psychology Glendon Campus York University Toronto, ON Canada; 2 Department of Psychology University of Windsor Windsor, ON Canada

**Keywords:** web-based testing, aging, cognition, neuropsychology, mobile phone

## Abstract

**Background:**

Web-based research allows cognitive psychologists to collect high-quality data from a diverse pool of participants with fewer resources. However, web-based testing presents unique challenges for researchers and clinicians working with aging populations. Older adults may be less familiar with computer usage than their younger peers, leading to differences in performance when completing web-based tasks in their home versus in the laboratory under the supervision of an experimenter.

**Objective:**

This study aimed to use a within-subjects design to compare the performance of healthy older adults on computerized cognitive tasks completed at home and in the laboratory. Familiarity and attitudes surrounding computer use were also examined.

**Methods:**

In total, 32 community-dwelling healthy adults aged above 65 years completed computerized versions of the word-color Stroop task, paired associates learning, and verbal and matrix reasoning in 2 testing environments: at home (unsupervised) and in the laboratory (supervised). The paper-and-pencil neuropsychological versions of these tasks were also administered, along with questionnaires examining computer attitudes and familiarity. The order of testing environments was counterbalanced across participants.

**Results:**

Analyses of variance conducted on scores from the computerized cognitive tasks revealed no significant effect of the testing environment and no correlation with computer familiarity or attitudes. These null effects were confirmed with follow-up Bayesian analyses. Moreover, performance on the computerized tasks correlated positively with performance on their paper-and-pencil equivalents.

**Conclusions:**

Our findings show comparable performance on computerized cognitive tasks in at-home and laboratory testing environments. These findings have implications for researchers and clinicians wishing to harness web-based testing to collect meaningful data from older adult populations.

## Introduction

### Background

The internet is an increasingly popular medium for running behavioral experiments in psychology [1-4]. In 2017, approximately a quarter of research papers in 4 top cognitive psychology journals featured at least one web-based study, up by 5% from the past 5 years [5]. This is an exciting paradigm shift for researchers given that web-based methods allow for the cost-effective collection of larger data sets from broader geographical regions and more diverse participants [6-10]. A growing number of studies have validated the use of web-based behavioral research by reproducing benchmark findings in cognitive psychology among web-based samples (eg, attentional blink, Flanker, Simon) [7] or finding equivalent performance between web-based and laboratory-tested samples on memory, perception, and attention tasks [8,11-15].

Cognitive aging research especially stands to reap the benefits of web-based testing: Participation among older adults may be hindered by restricted mobility and access to testing sites. As a result, many studies rely on convenience sampling where participants are self-selected due to the ease of recruitment and willingness to participate [16]. This is problematic as convenience samples of cognitively normal older adults tend to be younger and better educated than those recruited via population-based sampling [16-19] and more likely to have a family history of Alzheimer disease [17], all factors that can skew research findings. A similar issue exists in research on neurodegenerative diseases, where the research samples are overwhelmingly White, well-educated, and have a high socioeconomic status, limiting the generalizability of clinical research to the population at large [20].

An obstacle to web-based aging research is that adults aged above 65 years have lower rates of technology adoption than their younger peers [21] and unfamiliarity with computers may affect performance on computerized tasks. Moreover, Mechanical Turk by Amazon, the most popular crowdsourcing platform for psychology researchers, has a population of workers that tends to be younger than the overall population [22]. Nonetheless, some studies have examined cognitive abilities in large web-based samples with ages ranging from 10 to 70 years [23,24] and 10 to 85 years [25]. Web-based data collection has also been used to investigate age-related changes in prospective memory [26] as well as working memory and visuospatial processing [27]. More commonly, however, web-based research among older cohorts is often used to test the validity and reliability of web-based neuropsychological batteries for clinical purposes of cognitive screening [28] or tele-neuropsychology [29]. A small body of work in tele-neuropsychology has explored the use of web-based cognitive screens for self-monitoring of cognitive impairment [30-32]. There are promising avenues for better detection and monitoring of cognitive impairment using well-established cognitive tasks [33-35]. Nevertheless, most clinicians (ie, neurologists, neuropsychologists) continue to rely on paper-and-pencil testing conducted during in-office visits, using technology only sparingly in their assessments [36,37].

Given the ongoing effects of COVID-19 on health care delivery and behavioral research, there is a pressing need to establish and validate protocols for remote cognitive testing among older adults. A chief concern, however, is whether performance within a standard testing situation is comparable with testing done in an unsupervised web-based format [38]. Using a within-subjects design, Assman et al [39] found that a self-administered web-based cognitive battery (NutriCog) provided similar information to a version supervised by a neuropsychologist. However, they found learning effects such that performance was better on the second completion of the battery, independent of the mode of administration. A recent study by Backx et al [40] also used a within-subjects design to examine the effects of testing environment (supervised in the laboratory vs unsupervised at home) on performance on the Cambridge Neuropsychological Test Automated Battery. They found comparable performance across contexts, although reaction times (RTs) were slower in the web-based version. Although the results of these studies are encouraging, they did not examine older adults specifically. An advantage of supervised testing is that the neuropsychologist or experimenter can clarify instructions, provide encouragement, and ensure that the setting is free of distractions for participants—this may be especially important with older participants who are likely to be less fluent with technology. However, older adults may also be more stressed in such situations: novel testing locations have been shown to disproportionately stress older adults relative to younger adults, leading to greater age differences in memory [41].

### Objectives

The aim of this study is to investigate using a within-subjects design whether performance on computerized cognitive tasks differs as a function of the testing environment in a group of community-dwelling older adults aged above 65 years. We selected cognitive tasks that are well established in both experimental research and neuropsychology, have low susceptibility to practice effects, and are known to be sensitive to age-related changes. The word-color Stroop task [42] (response inhibition and processing speed) is a widely used test in both experimental psychology and clinical neuropsychology, with a large body of work demonstrating declines in Stroop performance due to normative aging [43] and age-related neurodegenerative disease [44]. Similarly, paired associates learning (PAL) has long been used as a measure of the associative nature of episodic memory, which is well-known to be affected during normative aging [45] and is strongly implicated in Alzheimer disease [46-48]. Participants completed computerized versions of these tasks in 2 testing sessions spaced 24 hours apart: unsupervised in their own home using their personal computer as well as supervised by an experimenter in the laboratory. If the testing environment does indeed affect performance on these web-based measures, we would predict a significant difference between scores across the 2 conditions: given the paucity of previous findings using these particular measures, we did not have any strong a priori hypothesis with respect to the directionality of effects of testing environment on performance (ie, performance would be better or worse in person vs on the web). In addition, we explored the extent to which scores on computerized cognitive tests correlated with their gold standard neuropsychological test equivalents and the extent to which performance on computerized tests is associated with technology use and familiarity.

## Methods

### Participant Recruitment

This study was powered to detect moderate effect sizes (Cohen *d*=0.50) at a power >0.80 (two-tailed α at .05). To date, no studies have compared these experimental measures across testing conditions among older adults; however, a handful of studies have compared performance on web-based neuropsychological tests as a function of testing location [28,39,40] and found moderate effects of testing location. A power analysis using G*Power 3 [49] determined that a sample size of 34 would be required to detect moderate effects (Cohen *d*=0.50) with a power >0.80 (two-tailed distribution with an =.5). A total of 38 adults age above 65 years were recruited via the York Research Participant Pool and agreed to participate in the study. The data of 6 participants were excluded: 3 due to computer-related issues and 3 due to participant error. The analyses included 32 participants (20 females). Participants were screened to ensure that they were diagnosed with any medical, neurological, or psychiatric condition known to impact cognition.

### Measures

#### Web-Based Cognitive Tasks

In total, 3 experimental tasks were completed on a computer. For the in-person testing session, the tasks were presented on a 23.8” Dell monitor and responses were provided on a QWERTY keyboard. The specifications of the computer used in the web-based testing session are unknown as participants used their personal devices. However, participants were told before being enrolled into the study that a QWERTY keyboard was required.

1. *Word-color Stroop task*: 36 congruent (eg, *blue* in blue ink) and 36 incongruent (eg, *blue* in yellow ink) stimuli were randomly presented to participants using PsyToolKit [31,32]. Participants were instructed to press the *r*, *y*, *g*, and *b* keys on the keyboard in response to words presented in red, yellow, green, and blue, respectively. If they did not respond within 4500 milliseconds, the following stimulus was presented. Participants first completed a practice trial with 6 trials before beginning the main task. Key outcome measures were raw RTs to respond to the congruent and incongruent trials, Stroop effects (calculated by subtracting RT to incongruent trials from RT to congruent trials), and errors (eg, pressing on the key corresponding to red when the ink was blue).
2. *PAL task*: 32 unrelated word pairs (eg, *baker-wagon*) were selected from the study by Connor et al [50] and divided into 2 sets of 16 pairs (set A and set B). There were no differences in word frequency or concreteness between sets, *F*_1,30_ <1. Stimuli were presented using Qualtrics. In total, 16 unrelated word pairs (eg, tool-coast) were randomly presented, one at a time, for 4 seconds followed by a 1-second interstimulus interval (study 1). Immediately after, participants completed a self-paced cued recall (eg, tool-?) for the word pairs they had just studied (immediate recall 1) using the keyboard to type their responses. The same study-test cycle was then repeated (study 2 followed by immediate recall 2). After a 15-min delay, they completed the delayed cued recall portion of the PAL task (eg, tool-?) at their own pace. Key outcome measures were a PAL learning score calculated by adding the number of correctly recalled words during immediate recall 1 and 2 as well as a PAL delayed memory score defined as the number of words recalled during the delayed cued recall.
3. *International Cognitive Ability Resource (ICAR)*: The ICAR is a public-domain cognitive assessment tool [51] that includes 4 item types measuring reasoning: three-dimensional rotation presents cube renderings and asks participants to identify which of the response choices is a rotation of the target stimulus. The letter and number items show participants a short digit or letter sequence and ask them to identify the next position in the sequence from among 6 choices. The matrix reasoning items present 3×3 arrays of geometric shapes with one of the 9 shapes missing, and participants are instructed to identify which of the 6 geometric shapes best complete the stimulus. Finally, the verbal reasoning items include logic questions. We created 2 sets of problems each with 4 items from each item type for a total of 16 questions per set (set A and set B). Stimuli were presented using Qualtrics. Participants were given 7.5 min to complete 4 verbal reasoning and 4 letter and number problems, followed by 7.5 min to complete 4 matrix reasoning and 4 three-dimensional rotation problems from the ICAR. All questions were in a multiple-choice format, and participants used the mouse to select their answer. The key outcome was total accuracy across verbal and matrix questions (score from 0 to 16).

#### Standardized Neuropsychological Tasks

The following neuropsychological tasks were administered in person by a research assistant. All testing was performed under the supervision of a licensed neuropsychologist (KR). The verbal Paired Associates subtest of the Weschler Memory Scale -IV (WMS-IV) and Color Word Interference test of the Delis-Kaplan Executive Function System (D-KEFS) were included so that we could compare performance with their computerized analogs (PAL and Stroop task, respectively). The Montreal Cognitive Assessment (MoCA), Patient Health Questionnaire-9 (PHQ-9), and Shipley Verbal subtest were included for the purposes of describing our sample and ensuring that participants did not exceed clinical cut-offs for cognitive impairment or depression.

1. Verbal Paired Associates subtest (WMS-IV) [52]: this test assesses the ability of an individual to learn unrelated word pairs. Participants were given the task according to standard instructions. Specifically, they were presented with 14 pairs of unrelated words at a rate of 1 pair every 3 seconds. They were then given the first word of each pair and asked to recall the second word. This was repeated for 4 trials using the same list of word pairs. After a delay of 15-min, participants were again given the first word of each pair and asked to recall the second word. Key outcomes include the total number of correctly recalled word pairs across the immediate recall trials (learning score) and the total number of words recalled after the delay (delayed score). These raw scores were then converted to age-corrected scaled scores.
2. Color Word Interference test (D-KEFS) [53]: participants were administered the color naming and interference conditions of this task according to standardized instructions. In the color naming condition, participants were shown a page of colored patches and had to name them one by one as fast as possible, without making mistakes. In the interference subtest, participants were shown a page with names of colors printed in various colors and were instructed for each word to name the color the word was printed in, rather than read the word itself. Participants were told to complete the task as quickly as possible without making mistakes. Key outcomes for both subtests were the time to completion (in seconds). These raw scores were then converted to age-corrected scaled scores.
3. MoCA [54]: this is a brief administered screening tool used to detect cognitive impairment. It assesses cognitive domains including short-term memory, visuospatial processing, executive functioning, attention, and orientation in time and space. The key outcome was the total score out of 30 (for geriatric samples, scores >26 are considered normal, whereas scores 18-25 indicate mild cognitive impairment, 10-17 indicate moderate cognitive impairment, and less than 10 indicate severe cognitive impairment).
4. PHQ-9 [55]: this is a self-administered 9-item measure of depression severity. The key outcome was the total score out of 27, with higher scores indicating greater depression severity.
5. The Shipley Verbal subtest (from the Shipley Institute of Living Scale) [56] was included as a brief measure of verbal abilities (scores range from 0-40, with higher scores reflecting greater ability). This test requires participants to identify synonyms for stimulus words presented in a multiple-choice format.

#### Computer Questionnaires

The 20-item Computer Anxiety Scale [57] and the 19-item Computer Anxiety Rating Scale [58] are questionnaires asking individuals to indicate their level of agreement (1: *strongly disagree* to 5: *strongly agree*) with statements pertaining to attitudes toward computer use (eg, *I feel apprehensive about using computers*). The Computer Aversion, Attitudes, and Familiarity Index [59] is a 40-item questionnaire that prompts participants to indicate the extent to which statements about computer use and feelings surrounding computers apply to them (−3: *absolutely false* to +3: *absolutely true*; eg, *I enjoy using computers*).

### Procedure

**Figure 1:**
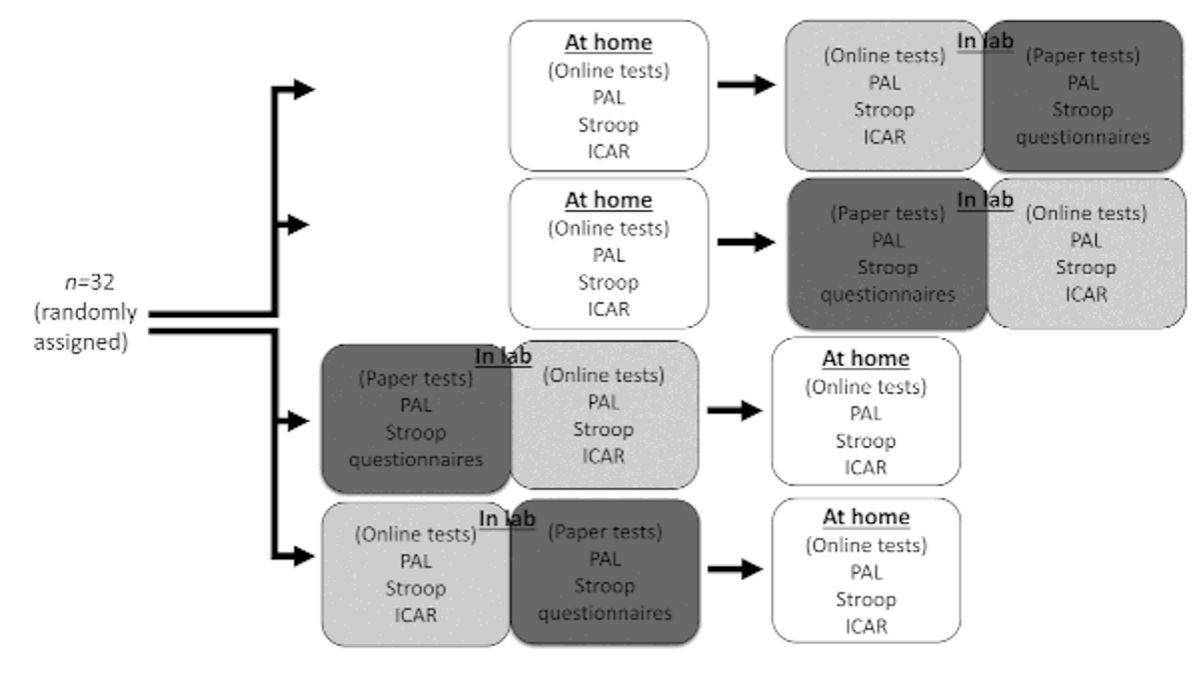
Flowchart of counterbalancing participant assignment to conditions in the experiment. ICAR: International Cognitive Ability Resource; PAL: paired associates learning.

All participants completed both an in-person testing session at the laboratory and a web-based testing session at their home, 24 hours apart. Whether participants completed the first testing session on the web (home first) or in-person (laboratory first) was counterbalanced across participants (Figure 1). The assignment of participants to order of testing (home first vs laboratory first) and order of test administration in the laboratory setting (web-based tests first vs paper-and-pencil tests first) was determined using a Latin square design. Upon recruitment, a participant was assigned to the next row in the Latin square, which determined their testing orders.

#### Web-Based Testing Session

Participants were sent a link to the study on the Qualtrics platform via email. After providing consent, they first completed the PAL task using stimuli from set A or B (set used was counterbalanced across participants). Finally, participants were redirected to the PsycToolkit site [60,61] to complete the word-color Stroop task. Participants were prompted to enter a 3-digit identifier before each task.

#### In-Person Testing Session

Participants completed 2 blocks of testing during the in-person session: an experimental testing block and a neuropsychological testing block. The order of the testing blocks was counterbalanced across participants. The experimental testing block was identical to the web-based testing session, with the exception that the PAL stimuli and the ICAR problems were different. For example, if a participant studied set A during the web-based testing session’s PAL task, they studied set B during the in-person testing session. Importantly, both the web-based testing and the in-person testing were completed on the PsycToolkit website, ensuring that the only difference between conditions was the testing location. In the neuropsychological testing block, participants completed the immediate and delayed recall conditions from the WMS-IV VPA (Verbal Paired Associates) test: the color naming condition and the interference condition from the D-KEFS Color Word Interference Test, MoCA, and the Shipley vocabulary test. In the 15-min interval between the VPA learning trials and the delayed cued recall, the following questionnaires were administered: PHQ-9, Computer Attitude Scale, Computer Anxiety Rating Scale, and Computer Familiarity Scale.

### Statistical Analyses

All analyses were conducted in Jamovi (version 1.2.27) using R statistical language, and an alpha level of .05 was used throughout.

To avoid the undue influence of extreme outliers on the Stroop task, trials where the participants responded under 200 milliseconds or over 4000 milliseconds were excluded. This led to 1.5% of total trials being excluded in the laboratory condition and 2% being excluded in the home condition. The Stroop data of 1 participant were removed in the home condition due to the fact that they missed all trials (RTs >4500 milliseconds).

Mixed analyses of variance were conducted to examine how performance on the outcome measures of our computerized experimental tasks (Stroop, PAL, and ICAR) varied as a function of testing environment (home vs laboratory) and testing order (home first vs laboratory first). In addition, Bayesian inferential testing was performed to provide a more comprehensive perspective on the equivalence of the test data across testing environments. This approach allows us to assign a probability of the null hypothesis or alternative hypothesis being true, given our obtained data [62]. Specifically, we conducted paired *t* tests and calculated corresponding Bayes factors for each *t* test using the BayesFactor R package [63] implemented in Jamovi to investigate the PAL total scores in both conditions (learning and delayed recall), mean RTs for Stroop (congruent, incongruent, and inhibition), and total scores on the ICAR reasoning task using testing environments as the paired conditions. The null hypothesis was defined as no meaningful difference in performance on these measures across testing environments, whereas the alternative hypothesis would be defined as a significant (nondirectional) difference in test scores between tasks done in the laboratory and on the web. As there are no prior studies on paired associate learning and Stroop task performance across in-laboratory and web-based settings, we did not have a strong a priori hypothesis regarding the presence or directionality of any effects of testing environment, other than a general alternative hypothesis of nonequivalence across testing conditions. In addition, given the lack of previous studies, we had no scientific knowledge to inform the most appropriate prior distribution. Thus, we used a Cauchy distribution centered around 0 (ie, the null) and specified a width parameter of 0.707. Results are presented in terms of a Bayes factor (BF01), which denotes the probability of the observed data, given the null hypothesis. Bayes factors were interpreted using the guidelines by Lee and Wagenmakers [64], which are as follows: Bayes factors below 1 are seen as evidence for the alternative hypothesis (0.33-1: anecdotal evidence, 0.1-0.33: moderate evidence; and <0.1 strong evidence), and Bayes factors above 1 are seen as evidence for the null hypothesis (1-3: anecdotal evidence, 3-10: moderate evidence; and >10 strong evidence).

To explore the validity of these experimental measures, Pearson correlations were conducted to explore the association between performance on the computerized experimental tasks and their pencil-and-paper analogs currently used in clinical practice. Specifically, we examined the relationship between performance on the computerized Stroop and the D-KEFS Color Word Interference Test as well as performance on the PAL task and the WMS-IV Verbal Paired Associates test. In addition, Pearson correlations were conducted to investigate the association between scores on the questionnaires querying computer attitudes, familiarity, and outcome measures on the computerized experimental tasks and the neuropsychological tests.

## Results

### Participant Characteristics

Demographic variables and neuropsychological scores as a function of testing session order are shown in Table 1.

**Table 1:** Mean demographic and neuropsychological scores as a function of order of testing environments.

Participant characteristics and neuropsychological variables		Order of testing environments, mean (SD)	
		Home first^a^	Laboratory first^b^
Age (years)		70.50 (6.87)	70.90 (7.30)
Years of education		17.90 (3.12)	17.90 (2.72)
PHQ-9^c^		2.56 (2.73)	1.00 (1.46)
MoCA^d^		27.70 (1.89)	27.30 (1.85)
Shipley		36.10 (3.90)	37.20 (1.47)
**WMS-IV^e^-Verbal Paired Associates**			
	Learning score (scaled score)	11.94 (2.46)	11.75 (2.96)
	Delayed score (scaled score)	11.81 (3.27)	11.19 (3.15)
**D-KEFS^f^-Color-Word Interference test (Stroop)**			
	Color naming (scaled score)	11.06 (2.46)	12.13 (2.36)
	Inhibition score (scaled score)	11.81 (3.10)	11.81 (1.72)

^a^Home testing session on day 1 and laboratory testing session on day 2.

^b^Laboratory testing session on day 1 and home testing session on day 2.

^c^PHQ-9: Patient Health Questionnaire-9.

^d^MoCA: Montreal Cognitive Assessment.

^e^WMS-IV: Wechsler Memory Scale-IV.

^f^D-KEFS: Delis-Kaplan Executive Functioning System Test.

The years of education of the 2 participants could not be confirmed. There were no significant differences in age (*t*_30_=0.15; *P*=.88) or years of education (*t*_28_=0.01; *P*=.99) as a function of session order. Participants assigned to the home-first testing order had marginally higher scores on the (PHQ-9) than those assigned to the laboratory-first testing order (*t*_30_=2.02; *P*=.05); however, none of the participants exceeded the clinical cut-off for major depressive disorder on the PHQ-9 (total score ≥10). There were no group differences in the MoCA (*t*_30_=0.57; *P*=.58) nor the Shipley vocabulary test (*t*_30_=1.02; *P*=.32). 

### Performance on Experimental Tasks Across Testing Environments: Frequentist Analyses

#### Stroop Task

We first conducted a 2 (Stroop condition: congruent vs incongruent)×2 (testing environment: home vs laboratory) repeated measures ANOVA with raw RTs as the dependent variable. RTs were significantly faster in congruent trials than incongruent trials (*F*_1,30_=54.54; *P*<.001; η^2^_p_=0.65), and there were no group differences in RTs across testing environments (*F*_1,30_=1.15; *P*=.29; η^2^_p_=0.04). The Stroop condition×testing environment interaction was not significant (*F*_1,30_<1; *P*=.77; η^2^_p_=<0.01). Next, we wanted to examine whether first being administered the Stroop test at home or in the laboratory would affect Stroop performance. A 2 (Stroop condition: congruent vs incongruent)×2 (order of testing environment: home first vs laboratory first) mixed ANOVA with reaction time on the Stroop test completed at home revealed a significant effect of condition (*F*_1,30_=81.33; *P*<.001; η^2^_p_=0.73) and no order effect (*F*_1,30_=1.46; *P=*.24; η^2^_p_=0.05). The interaction was insignificant (*F*_1,30_<1; *P*=.43; η^2^_p_=0.02). The same analysis as above was conducted but with RT on the Stroop test completed in the laboratory. Participants were faster on congruent trials than incongruent trials (*F*_1,30_=30.40; *P*<.001; η^2^_p_=0.51), and there was no order effect (*F*_1,30_<1; *P*=.52; η^2^_p_=0.01). The interaction was insignificant (*F*_1,30_<1; *P=*.59; η^2^_p_=0.01).

We repeated the set of analyses above to examine Stroop errors as a function of testing environments and testing order. A 2 (Stroop condition: congruent vs incongruent)×2 (testing environment: home vs laboratory) repeated measures ANOVA with errors on the Stroop test as the dependent variable revealed that participants made more errors on the incongruent compared with congruent trials (*F*_1,30_=11.33; *P=*.002; η^2^_p_=0.27). There was no significant main effect of the testing environment (*F*_1,30_<1; *P=*.55; η^2^_p_=0.01), and the Stroop condition×testing environment interaction was insignificant (*F*_1,30_=2.41; *P*=.13; η^2^_p_=0.07). A 2 (Stroop condition: congruent vs incongruent) ×2 (order of testing environment: home first vs laboratory first) mixed ANOVA with errors on the Stroop test completed at home revealed a significant effect of condition (*F*_1,30_=90.94; *P*=.004; η^2^_p_=0.25) and no order effect (*F*_1,30_=1.99; *P*=.17; η^2^_p_=0.06). The interaction was insignificant (*F*_1,30_=1.29; *P*=.27; η^2^_p_=0.04). The same analysis as above was conducted but with errors committed on the Stroop test completed in the laboratory. Errors were equivalent across conditions (*F*_1,30_=1.45; *P*=.24; η^2^_p_=0.05) and the order of testing environment main effect was insignificant (*F*_1,30_=2.28; *P*=.14; η^2^_p_=0.07). The interaction was insignificant (*F*_1,30_<1; *P*=.87; η^2^_p_<0.01).

Finally, Stroop effects were calculated for each participant by subtracting the RT for congruent trials from the RT for incongruent trials. We then conducted a 2 (testing environment: home vs laboratory)×2 (order of testing environment: home first vs laboratory first) mixed ANOVA with these Stroop effect scores as the dependent variable. The results showed no significant main effect of the testing environment (*F*_1,29_<1; *P*=.78; η^2^_p_<0.01) or order of testing environment (*F*_1,29_<1; *P*=.45; η^2^_p_=0.02). The testing environment×order of testing environment interaction was insignificant (*F*_1,29_<1; *P*=.89; η^2^_p_<0.01).

#### PAL

We conducted a 2 (testing environment: home vs laboratory)×2 (order of testing environment: home first vs laboratory first) mixed ANOVA with PAL learning scores as the dependent variable (Table 2).

**Table 2:** Mean performance on experimental tasks as a function of testing environment and order of testing environment (SDs in parentheses).

Variables		Home testing session, mean (SD)		Laboratory testing session^a^, mean (SD)	
		Home first^b^	Laboratory first^c^	Home first	Laboratory first
**Word-color Stroop**					
	Congruent—RT^d^ (ms)	1415.63 (289.03)	1317.13 (253.14)	1276.61 (317.03)	1374.46 (315.87)
	Incongruent—RT (ms)	1661.52 (320.55)	1522.54 (280.04)	1510.95 (461.22)	1566.58 (257.71)
	Interference scores (ms)	245.89 (168.18)	205.41 (108.55)	234.34 (216.48)	192.12 (213.85)
	Congruent—errors	0.43 (1.32)	0 (0)	0.50 (2.00)	0.13 (0.34)
	Incongruent—errors	1.50 (2.63)	0.50 (1.27)	0.44 (1.09)	0.63 (0.89)
**Paired associates learning**					
	Learning scores	18.13 (8.28)	17.25 (8.56)	15.75 (8.41)	15.06 (9.73)
	Delayed cued recall	10.68 (4.54)	9.94 (4.72)	9.88 (4.80)	9.13 (5.24)
ICAR^e^ scores		5.75 (3.21)	6.88 (2.31)	5.69 (1.96)	5.75 (2.54)

^a^Laboratory testing session on day 2.

^b^Home testing session on day 1 and laboratory testing session on day 2.

^c^Laboratory testing session on day 1 and home testing session on day 2.

^d^RT: reaction time.

^e^ICAR: International Cognitive Ability Resource.

Results showed no significant difference in cued recall learning scores across testing environments (*F*_1,30_=3.57; *P*=.07; η^2^_p_=0.106) or as a function of the order of testing environment (*F*_1,30_<1; *P*=.79; η^2^_p_<0.01). The testing environment×order of testing environment interaction was insignificant (*F*_1,30_<1; *P*=.94; η^2^_p_<0.01).

Next, we conducted the same analysis as above, with delayed cued recall scores as the dependent variable. There was no effect of testing environment (*F*_1,30_=1.66; *P*=.21; η^2^_p_=0.05) or order of testing environment (*F*_1,30_<1; *P*=.64; η^2^_p_<0.01). The interaction was insignificant (*F*_1,30_<1; *P*=.99; η^2^_p_<0.01).

#### ICAR

We ran a 2 (testing environment: home vs laboratory)×2 (order of testing environment: home first vs laboratory first) mixed ANOVA with ICAR scores as the dependent variable (Table 2). This showed insignificant main effects of testing environment (*F*_1,30_=1.55; *P*=.22; η^2^_p_=0.05) and order of testing environment (*F*_1,30_<1; *P*=.44; η^2^_p_=0.04). The interaction was insignificant (*F*_1,30_=1.24; *P*=.28; η^2^_p_=0.04).

### Performance on Computerized Tasks Across Testing Environments: Bayesian Analyses

The Bayesian paired samples *t* test of PAL learning scores yielded a Bayes factor of 1.04, indicating that the data could be consistent with either the null hypothesis or the alternative hypothesis. However, on PAL delayed recall, there was a Bayes factor of 2.44, providing anecdotal evidence that data were 2.44 times more likely under the null hypothesis (ie, the groups of test scores were equivalent across testing environments). In terms of Stroop RT performance, results from the paired *t* test for the congruent condition indicated that the data were 3.4 times more likely under the null hypothesis than the alternative hypothesis (BF01=3.40). Similarly, RTs from the incongruent condition and the Stroop effects (incongruent RT-congruent RT) also provided moderate evidence that the null hypothesis was more likely than the alternative hypothesis (BF01=3.12 and 4.34, respectively). Finally, the Bayesian paired *t* test on the ICAR reasoning total scores yielded a Bayes factor of 2.63, providing anecdotal evidence that the data were more likely under the null hypothesis than the alternative hypothesis. Collectively, these results bolster the notion that there was no meaningful difference in performance on computerized PAL, Stroop, and ICAR reasoning tasks when done in a laboratory or on the web. Prior and posterior distribution plots and Bayes factor robustness checks are provided in Multimedia Appendix 1.

### Correlations Between Computerized Tasks and Standard Neuropsychological Tests

Regarding Stroop performance, we found no significant correlation between mean RT for the congruent condition and color naming on the D-KEFS Color Word Interference Test (*r*=0.13; *P*=.47; 95% CI −0.23 to 0.46). However, we did find a significant positive association between mean RT in the incongruent condition and the inhibition subtest (*r*=0.69; *P*<.001; 95% CI 0.46 to 0.84). We found a similar significant positive association between PAL total learning scores across 2 trials and the total learning score on the WMS-IV Verbal Paired Associates test (*r*=0.67; *P*<.001; 95% CI 0.42 to 0.83). In terms of delayed recall, there was also a significant positive association (*r*=0.67; *P*<.001; 95% CI 0.41 to 0.82). Collectively, these findings suggest a robust association between performance on web-based computerized tests and standard neuropsychological tests completed in person.

### Test-Retest Reliability of Web-Based Cognitive Measures

We also conducted intraclass correlations between PAL and Stroop scores obtained at home and in the laboratory to obtain an estimate of the reliability of these measures over time. Regarding the Stroop test, there were adequate ICC (intraclass correlations) values between scores obtained in the laboratory and on the web for the congruent (*r=*0.72; *P*<.001; 95% CI 0.49 to 0.85) and incongruent (*r=*0.75; *P*<.001; 95% CI 0.53 to 0.87) conditions. The ICC for the interference condition was modest (*r=*0.61; 95% CI 0.34 to 0.79). For PAL, there were adequate ICC values between scores obtained in the laboratory and on the web for the total learning score (*r=*0.70; *P*<.001; 95% CI 0.46 to 0.84) and delayed recall score (*r=*0.73; *P*<.001; 95% CI 0.51 to 0.86) conditions.

### Correlations Between Computerized Tasks and Computer Questionnaires

Scores on the 3 questionnaires (ie, Computer Anxiety Scale [CAS], Computer Anxiety Rating Scale [CARS], and Computer Aversion, Attitudes, and Familiarity Index [CAAFI]) were scored for each participant. The mean scores and correlations among the questionnaires are shown in Table 3.

**Table 3:** Pearson correlations among questionnaires.

Questionnaires	Mean (SD)	1		2	
		Correlation coefficient	*P* value	Correlation coefficient	*P* value
1. CAAFI^a,b^	−11.20 (13.30)	—^c^	—	—	—
2. CARS^d,e^	40.30 (14.00)	−0.649	<.001	—	—
3. CAS^e^	70.10 (10.10)	0.487	.005	−0.584	<.001

^a^CAAFI: Computer Aversion, Attitudes, and Familiarity Index.

^b^Higher scores on the CAAFI reflect greater familiarity and more positive attitudes toward computers.

^c^Correlation scores not applicable.

^d^CARS: Computer Anxiety Rating Scale.

^e^Higher scores on CARS and the Computer Anxiety Scale (CAS) reflect lesser and greater computer-related anxiety, respectively.

Questionnaire scores did not differ as a function of the order of the testing environment for the CAS (*F*_1,30_=3.31; *P*=.08), CARS (*F*_1,30_=1.80; *P*=.20), or CAAFI (*F*_1,30_<1; *P*=.77). There were significant correlations between CAS and CARS scores and ICAR scores completed at home (*r=*0.50, *P*=.004 and *r=*−0.45, *P=*.01, respectively). However, there were no significant correlations between these measures when completed in the laboratory nor were there any other significant correlations between scores on any of the questionnaires and performance on the computerized tasks (Multimedia Appendix 1).

## Discussion

### Principal Findings

The primary aim of this study is to examine whether performance on computerized versions of well-known cognitive tasks (ie, word-color Stroop, PAL, and matrix and verbal reasoning) would vary as a function of the testing environment (supervised in the laboratory vs unsupervised at home) among healthy older adults. Our results align with other studies that found comparable results across testing environments using a within-subjects design [39,40] and extend them to older adults. Our findings are encouraging for researchers and clinicians looking to harness web-based testing among older adult populations. We found no significant differences in performance on any of the computerized tasks across testing environments, a pattern of results supported by complementary Bayesian analyses. Crucially, there were no order effects, that is, whether participants completed the at-home or in-person testing session first had no influence on performance. There was no consistent correlation between the measures of computer familiarity or attitudes and performance on any of the computerized tasks. This is congruent with past research finding that computer familiarity did not mediate benefits derived from web-based memory training [65]. There is some evidence, however, that the total learning score on PAL may not be equivalent across contexts, given the *P* value approached significance and the Bayesian analysis indicated that the data were not more consistent with either the null hypothesis or alternative hypothesis. Further studies are required to replicate this finding and establish a more precise estimate of any putative differences due to the testing location. It is interesting to note that the scores obtained during web-based testing (ie, in the participant’s home) were higher on average than those obtained in the laboratory, which is counterintuitive to the idea that performance should suffer in an uncontrolled environment with more potential distractors. Nevertheless, the results indicate that older adults can produce equivalent results on tests tapping into various cognitive domains, regardless of whether they are done at home or in the laboratory.

Our findings are reassuring for experimental researchers seeking to extend their web-based research program to older adult populations. Our findings support the viability of testing older adults in their homes, which is likely a lower stress environment than a laboratory or office [41]. Past studies have found that older adults report preferring computerized over traditional assessments [66] and that they value being able to choose the timing [67] and circumstances [68] of at-home assessments. Our findings also have relevance for clinical neuropsychology, a field that has been slow to integrate technology into practice [36]. Although our study was among cognitively healthy adults, the fact that we found equivalent task performance on several cognitive tests across testing environments supports the further investigation and validation of computerized measures in geriatric patients, which can open new avenues for the diagnosis and monitoring of cognitive functioning. Adapting experimental paradigms into clinical assessment protocols may prove useful for increasing precision in measuring underlying cognitive constructs (ie, validity) and in drawing brain-behavior associations [69]. Important next steps would be to validate web-based testing as an appropriate means to measure cognition to support diagnosis and also as an appropriate assay of everyday functioning in key cognitive domains such as memory [70], given that age differences in memory tend to be minimized in the real world relative to laboratory settings [71].

The need for further research into the utility of remote testing has been brought to the forefront by the ongoing COVID-19 pandemic. Much of the extant work has focused on administering existing cognitive screens and neuropsychological tests via tele-conferencing [29,39,66,72,73] rather than exploring updated options, such as using well-validated experimental tasks in a clinical context. Looking into the future, incorporating data collected from wearables, smartphone apps, and/or other sensors may also provide a rich source of data for better detection and monitoring of cognitive [37,74] and mood symptoms in neurodegenerative diseases [75]. For example, if some cognitive domains can be reliably measured using web-based cognitive tasks with acceptable psychometric properties [28,76-78], clinical practice can shift toward more remote monitoring of cognitive changes in memory or executive functioning, given that these domains are key factors in the loss of functional independence in neurodegenerative diseases [79].

An additional, encouraging finding regarding the validity of these computerized measures is that participants’ performance on web-based computerized cognitive tasks was significantly associated with performance on analogous standard neuropsychological tests, with correlations in the order of 0.6, and CIs showing a lower-bound correlation of approximately 0.4. These findings suggest that across a sample of healthy older adults, the rank order of their performance on standard neuropsychological tests is generally preserved when examining web-based test scores. However, unlike the Stroop and the PAL tasks, we did not include a paper-and-pencil analog for our computerized ICAR task, so we could not estimate its validity with current clinical tools. Although subsequent research is needed with more robust samples, these preliminary results are consistent with a recent study [75] showing that normative data from web-based measures can be used for individual differences research and eventually to guide decision making about individual patients.

### Strengths and Limitations

Our study examined cognitive task performance across at-home and in-laboratory settings within the same group of older adults. A limitation of our study is that participants were recruited via a university participant pool. As discussed above, it is likely that participants recruited via population-based sampling would be lower in education and higher in age, which would likely yield lower familiarity with computer usage. However, it is important to note that over time, older cohorts will be increasingly technology savvy, so this will not be an enduring issue: 67% of adults aged above 65 years report going on the internet, up from 13% in the early 2000s, and the figure increases to 82% when we look at the youngest-old between the ages of 65 and 69 years [21]. Our study also had participants performing the web-based tasks on different devices as the at-home computer was their own. Although this was not an issue for our purposes, future research should consider using the same devices, especially for screening and diagnosis. Finally, it should be noted that 3 participants (9% of our sample) had to be excluded due to user problems. Our study required individuals to navigate to 2 different platforms to complete the tasks, which may have added confusion. Improving the design of computerized tasks continues to be an important goal for bringing cognitive testing on the web.

### Conclusions

In summary, we provide evidence that healthy older adults who conduct computerized cognitive tests on a web-based platform can produce results comparable with those obtained in a laboratory environment. Moreover, performance on these web-based measures was correlated with standard neuropsychological test performance but was not correlated with technology familiarity. The results serve as a starting point for future studies on the validity of web-based platforms for measuring cognition in healthy and unhealthy aging populations.

## Appendix

Multimedia Appendix 1 Supplementary correlational matrix and output of Bayesian analyses.

## Abbreviations

CAAFI: Computer Aversion, Attitudes, and Familiarity Index
CARS: Computer Anxiety Rating Scale
CAS: Computer Anxiety Scale
D-KEFS: Delis-Kaplan Executive Function System
ICAR: International Cognitive Ability Resource
ICC: intraclass correlations
MoCA: Montreal Cognitive Assessment
PAL: paired associates learning
PHQ-9: Patient Hospital Questionnaire 9
RT: reaction time
VPA: Verbal Paired Associates
WMS-IV: Weschler Memory Scale -IV
