# Supplementary material for: Web-Based Cognitive Testing of Older Adults in Person Versus at Home: Within-Subjects Comparison Study
Source: JMIR Aging. 2021 Feb 1;4(1):e23384. doi: 10.2196/23384 (PMC8081157; doi:10.2196/23384)
Supplement: Multimedia Appendix 1 [file aging_v4i1e23384_app1.docx]

# Supplementary Materials

Pearson correlations between self-report measures of computer-related anxiety and computerized cognitive tasks completed at home.

|  | CAS | CARS | CAAFI |
| --- | --- | --- | --- |
| PAL Learning | -0.21 | -0.14 | 0.09 |
| PAL Delay | -0.08 | -0.14 | 0.08 |
| ICAR | 0.50** | -0.45* | 0.21 |
| Stroop Congruent | -0.08 | 0.31 | -0.12 |
| Stroop Incongruent | -0.04 | 0.27 | 0.03 |
| *Note*. * *p* < 0.05, ***p* < 0.01. PAL = Paired Associate Learning; ICAR = International Cognitive Ability Resource; CAS = Computer Anxiety Scale; CARS = Computer Anxiety Rating Scale; CAAFI = Computer Aversion, Attitudes, and Familiarity Index. Higher scores on the CAAFI reflect greater familiarity and more positive attitudes towards computers. Higher scores on the Computer Anxiety Scale (CAS) and the Computer Anxiety Rating Scale (CARS) reflect lesser and greater computer-related anxiety, respectively. | | | |

Prior and Posterior distribution plots and Bayes Factor robustness checks for Bayesian paired samples t-tests comparing test scores obtained online vs. in-lab


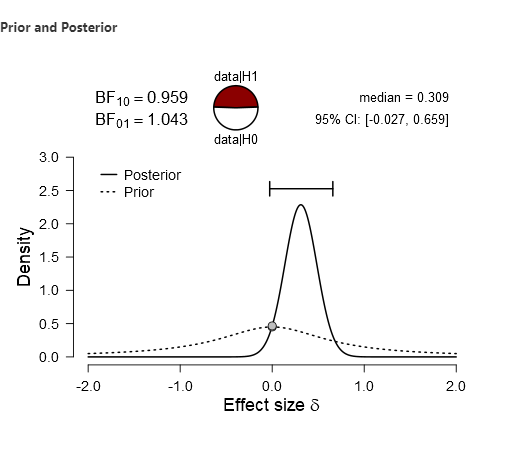
PAL total learning scores:


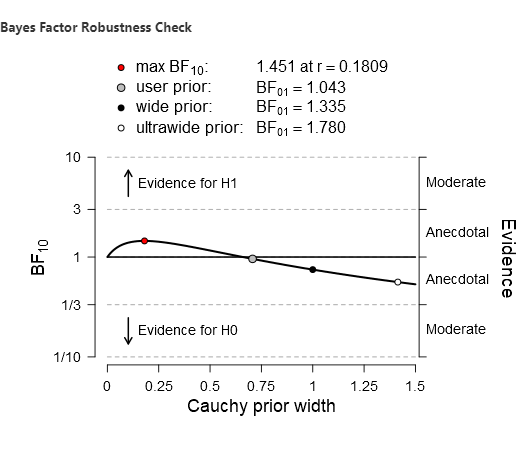


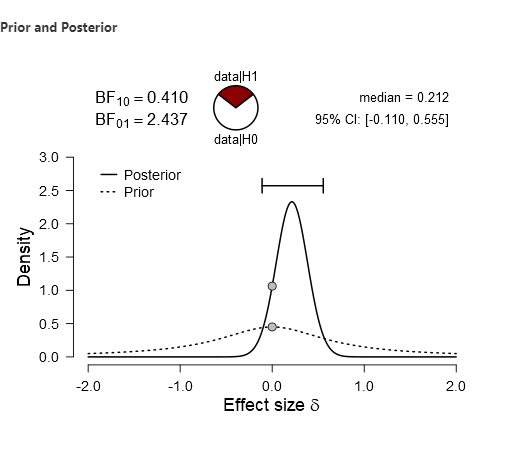
PAL delayed recall scores


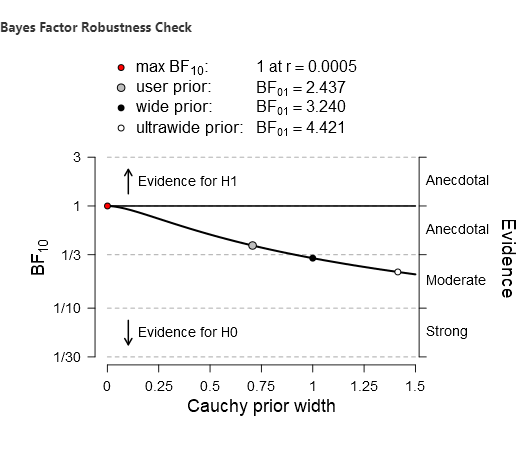


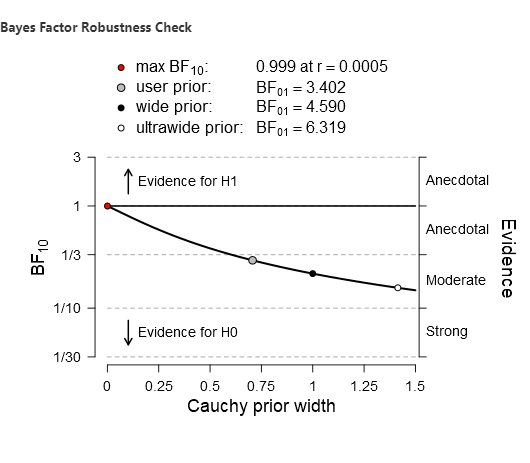

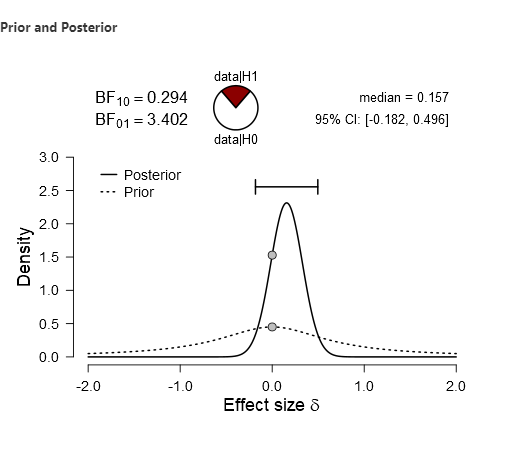
Stroop congruent condition RT

Stroop incongruent condition RT


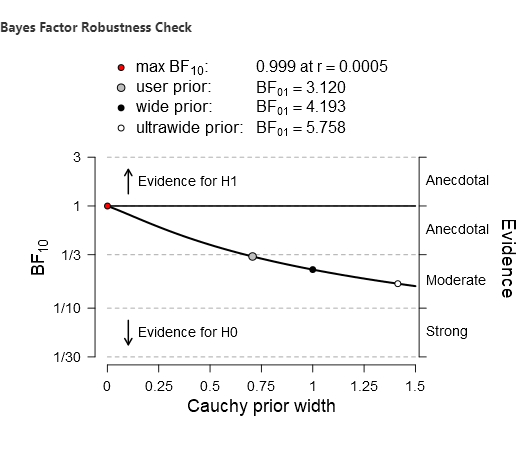


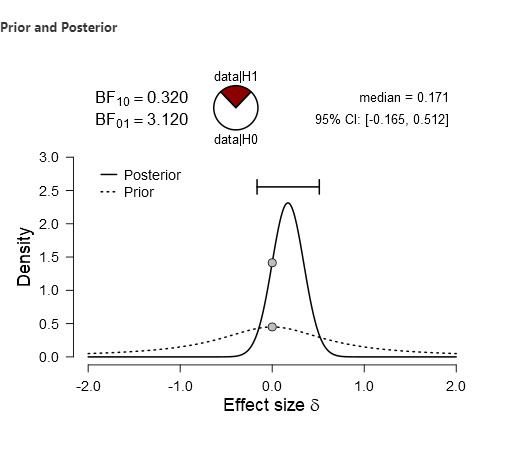


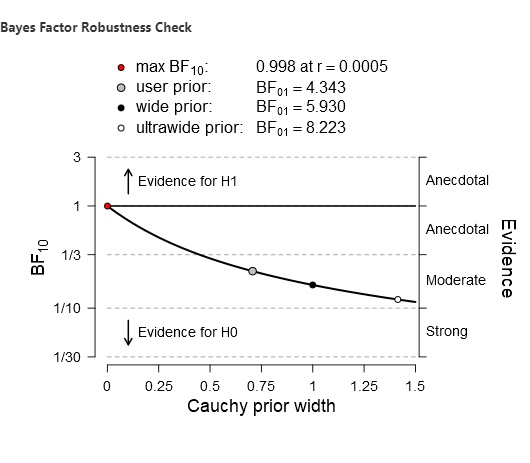

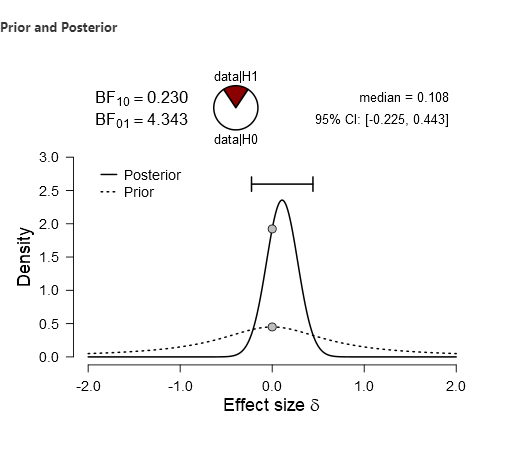
Stroop interference condition RT


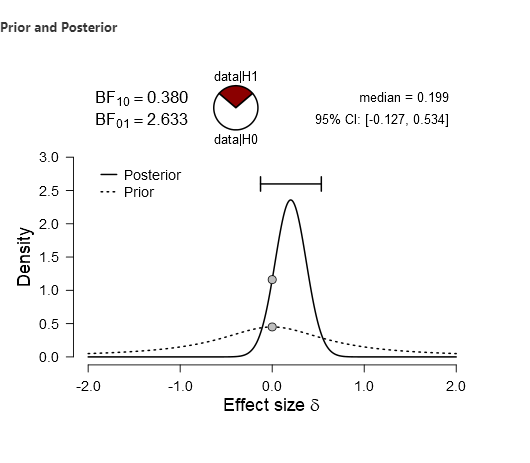

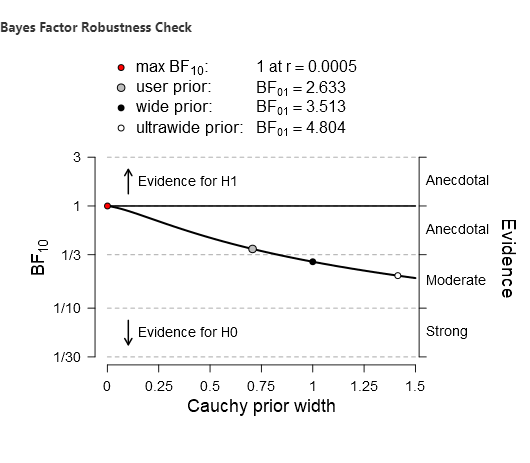
ICAR scores
